# Shanyu Wan attenuates age-related muscle alterations in SAMP8 mice with improved mitochondrial function

**DOI:** 10.1016/j.jtcme.2026.07.002

**Published:** 2026-07-22

**Authors:** Yue Hu, Qing Xiang, Cai Jiang, Chunxiu Huang, Huanghao Zhou, Yanyan Lan, Huijuan Wu, Zuanfang Li, Xiaodan Wang, Cong Chen, Lidian Chen

**Affiliations:** a Rehabilitation Industry Institute, Fujian University of Traditional Chinese Medicine, Fuzhou, 350122, China; b National-Local Joint Engineering Research Center of Rehabilitation Medicine Technology, Fujian University of Traditional Chinese Medicine, Fuzhou, 350122, China; c College of Rehabilitation Medicine, Fujian University of Traditional Chinese Medicine, Fuzhou, Fujian, 350122, China; d Innovation and Transformation Center, Fujian University of Traditional Chinese Medicine, Fuzhou, Fujian, 350122, China

**Keywords:** Shanyu Wan, Sarcopenia, Muscle disorders, Mitochondrial function, PGC-1α

## Abstract

**Background and aim:**

The traditional Chinese formula Shanyu Wan (SYW), comprising *Panax ginseng*, *Dioscorea opposita Thunb* and *Atractylodes Rhizoma*, has been empirically employed for metabolic regulation, although its mechanistic basis remains incompletely elucidated.

**Experimental procedure:**

Senescence-accelerated mouse prone 8 (SAMP8) mice were orally administered SYW by gavage at 2.56 g/kg for 8 weeks, with age-matched senescence-accelerated mouse resistant 1 (SAMR1) mice serving as controls. Muscle phenotype was evaluated by forelimb grip strength, rotarod performance, spontaneous locomotor activity, and magnetic resonance imaging (MRI). Histopathological alterations were evaluated by Masson's trichrome staining. Mitochondrial ultrastructure was examined by transmission electron microscopy (TEM), and mitochondrial functional parameters were subsequently determined. Furthermore, the effects of SYW-containing serum on oxidative injury were examined in *t*-BHP-treated C2C12 myoblasts, together with pharmacological interference analysis of PGC-1α-related signaling.

**Results and conclusion:**

Oral administration of SYW significantly improved muscle volume, grip strength, and motor performance in SAMP8 mice. These protective effects were accompanied by partial restoration of mitochondrial ultrastructure, increased mitochondrial respiratory chain complex activity, reduced oxidative stress, and changes in PGC-1α-related signaling. *In vitro*, SYW-containing serum attenuated *t*-BHP-induced oxidative and mitochondrial injury in C2C12 myoblasts, while pharmacological interference experiments suggested the involvement of PGC-1α-related signaling. These findings provide preclinical evidence that SYW may alleviate age-related muscle alterations by improving mitochondrial function and redox balance.

## Highlights

• Shanyu Wan improved sarcopenia-like phenotype in SAMP8 mice.
• Shanyu Wan alleviated mitochondrial damage and respiratory dysfunction.
• Shanyu Wan reduced oxidative stress and enhanced Nrf2 signaling.
• PGC-1α-related signaling may contribute to Shanyu Wan-mediated protection.

## Graphical abstract

**Figure:**
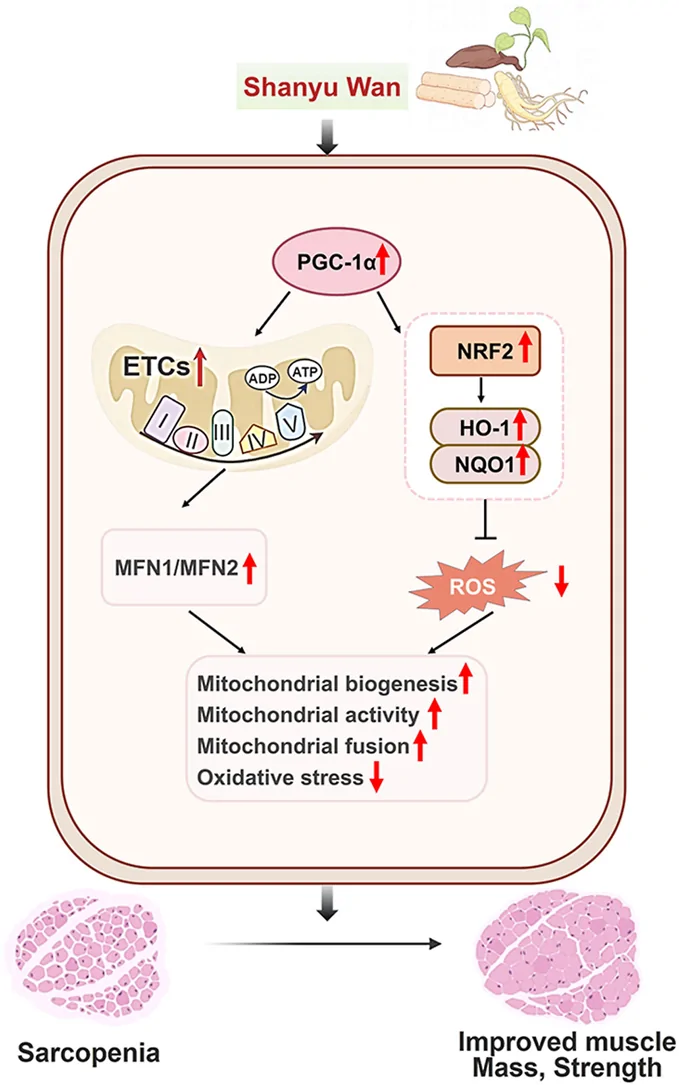

## 1 Introduction

Population aging has become a defining demographic trend worldwide, with the proportion of individuals over 65 years projected to reach 20% by 2050.^1^ While human longevity has improved, the accompanying decline in physiological function has led to a higher prevalence of chronic diseases and age-associated syndromes.^2^ Among these conditions, sarcopenia, defined as the progressive loss of skeletal muscle mass, strength, and physical performance, is a major contributor to frailty, functional dependence, and reduced quality of life in older adults.^3^, ^4^, ^5^ Understanding the molecular mechanisms underlying sarcopenia and identifying effective therapeutic strategies are therefore crucial for improving geriatric health.

Age-related sarcopenia is a multifactorial disorder involving metabolic dysregulation, impaired proteostasis, defective regenerative capacity, and structural remodeling of skeletal muscle.^6^ Among these mechanisms, mitochondrial dysfunction is increasingly recognized as a central driver of muscle aging.^7^^,^^8^ Disrupted mitochondrial homeostasis in sarcopenia involves oxidative imbalance, altered mitochondrial dynamics, defective quality control, and maladaptive stress signaling, all of which contribute to impaired contractile function and progressive muscle degeneration.^9^ These advances support mitochondrial homeostasis as a mechanistically relevant therapeutic target in age-related muscle dysfunction.

Peroxisome proliferator-activated receptor gamma coactivator 1-alpha (PGC-1α) is a key regulator of mitochondrial function and metabolic adaptation in skeletal muscle.^10^^,^^11^ As a transcriptional coactivator, PGC-1α coordinates the expression of nuclear-encoded mitochondrial genes and is closely linked to oxidative metabolism, mitochondrial biogenesis, and adaptive remodeling of myofibers.^12^^,^^13^ Recent work has further emphasized that PGC-1α-related signaling contributes not only to mitochondrial maintenance but also to broader metabolic adaptations of skeletal muscle under physiological and pathological conditions.^14^^,^^15^ Accordingly, interventions capable of preserving or restoring PGC-1α-related mitochondrial regulation may have therapeutic value in sarcopenia.

Current evidence-based management of sarcopenia primarily relies on physical exercise and nutritional support.^16^, ^17^, ^18^ In particular, physical activity is widely recognized as a core intervention because it improves skeletal muscle metabolic function, mitochondrial oxidative capacity, and redox homeostasis. However, exercise-based strategies may be difficult to implement in frail or mobility-impaired older adults, highlighting the need for complementary interventions that target related biological processes through non-exercise-based mechanisms. Traditional Chinese medicine (TCM) formulations, characterized by multi-component and multi-target actions, have shown potential benefits in improving muscle function and metabolic homeostasis.^19^^,^^20^ Shanyu Wan (SYW), a classical TCM formula composed of *Panax* g*inseng*, *Dioscorea opposita Thunb* and *Atractylodes Rhizoma*, has traditionally been used to improve vitality and physical function. Experimental evidence indicates that *Panax* g*inseng* can improve muscle performance,^21^^,^^22^ whereas the roles of *Dioscorea opposita Thunb* and *Atractylodes Rhizoma* in age-related skeletal muscle degeneration remain less clearly defined. Given its multi-component composition, SYW may exert synergistic effects on mitochondrial homeostasis and redox regulation, although its role in age-related skeletal muscle degeneration remains insufficiently defined.

To address this question, we used 8-month-old senescence-accelerated mouse prone 8 (SAMP8) mice as an accelerated-aging model of early sarcopenia-like muscle decline, characterized by impaired muscle performance together with mitochondrial dysfunction and oxidative imbalance.^23^^,^^24^ Because this model reflects accelerated senescence rather than natural human sarcopenia, it should be considered a mechanistically relevant preclinical model rather than a complete clinical surrogate.^25^ In the present study, we therefore investigated whether SYW could ameliorate sarcopenia-like muscle alterations by improving mitochondrial homeostasis and redox balance. Using SAMP8 mice and *t*-BHP-treated C2C12 cells, we evaluated the effects of SYW on muscle phenotype, mitochondrial ultrastructure, mitochondrial function, antioxidant responses, and their association with PGC-1α-related signaling. Thus, the present study addresses an important gap between established exercise-related mitochondrial adaptations and the potential of a complementary herbal intervention to modulate related mechanisms in age-related muscle dysfunction.

## 2 Materials and methods

### 2.1 Materials and reagents

All herbal components of SYW were obtained from certified suppliers and authenticated according to the Chinese Pharmacopoeia (2020 edition). The formula consisted of *Dioscorea opposita Thunb*, *Panax ginseng*, and *Atractylodes Rhizoma* in a weight ratio of 4:3:4. Dried raw herbs were pulverized into fine powder and suspended in a 1:5 (*w/v*) rice soup prior to oral administration. SYW was administered at doses of 0.64, 1.28, or 2.56 g/kg/day by gavage. The dosage range was established based on preliminary dose-screening experiments and body surface area conversion from the conventional clinical dosage.

### 2.2 Animals and treatments

Ten 3-month-old male senescence-accelerated mouse resistant 1 (SAMR1) SPF mice and forty age-matched senescence-accelerated mouse prone 8 (SAMP8) SPF mice were obtained from Huafukang Biotechnology (Beijing, China). At 8 months of age, hindlimb muscle volume, forelimb grip strength and rotarod performance were assessed to verify sarcopenia-like changes in SAMP8 mice. SAMP8 mice showed significant reductions in hindlimb muscle volume, grip strength and rotarod latency compared with age-matched SAMR1 mice, as presented in Fig. S1. The SYW dose was determined from the conventional human dosage using body surface area conversion. Based on the average pill weight and routine clinical dosage, the mouse-equivalent dose was calculated as 1.28 g/kg/day and defined as the medium dose, whereas 0.64 and 2.56 g/kg/day were used as the low and high doses, respectively. SYW was suspended in rice soup as the vehicle for oral gavage and administered at a total daily volume of 0.01 mL/g body weight, divided into two equal doses. Vehicle-treated SAMP8 mice received the same volume of rice soup according to the same schedule. SAMP8 mice were randomly assigned to model, low-, medium-, and high-dose groups, corresponding to 0, 0.64, 1.28, and 2.56 g/kg/day, respectively, and all treatments were continued for 8 weeks.

Before and after intervention, hindlimb muscle volume and forelimb grip strength were assessed. Motor coordination and endurance were evaluated using the rotarod test (Med Associates, USA). Spontaneous locomotor activity was monitored for 24 h using a computerized activity wheel system (Med Associates, USA) connected to an external sensor, with running distance and activity level recorded automatically. After treatment, mice were euthanized under isoflurane anesthesia, and skeletal muscle tissues were collected for subsequent analyses. All mice experiments were approved by the Animal Care and Use Committee of Fujian University of Traditional Chinese Medicine (FJTCM IACUC 2022123).

### 2.3 Morphological imaging

Structural *in vivo* MR images of mouse hind limbs were acquired via a 7T scanner (Bruker BioSpec 70/20, Germany). During scanning, the mice were anaesthetized with isoflurane in an oxygen mixture (95% O_2_ + 5% CO_2_) via a nasal catheter and positioned in a stereotaxic cradle. Multiple-slice RARE (rapid acquisition with relaxation enhancement) sequences were used to acquire conventional T2-weighted images (T2WIs) to obtain detailed hind limb muscle images. A 38 mm quadrature volume coil was used for limb detection. The magnetic resonance imaging (MRI) settings were as follows: echo time of 17 ms, repetition time of 2000 ms, 2 averages, 1 repetition, a field of view of 35 × 35 mm, an image resolution of 256 × 256 pixels, 40 slices, and a slice thickness of 0.6 mm. For volume measurements, the raw data were processed via ITK-Snap software. The volume of each muscle layer was calculated as the product of its area and slice thickness (0.6 mm). The total hind limb volume was determined by summing the volumes of all muscle layers. The muscle layers were semiautomatically segmented via the ITK-Snap snake tool (v3.8.0), and the total volume of each hind limb was the aggregate of all the segmented layers.

### 2.4 Morphological analysis

The gastrocnemius and soleus muscles were collected and fixed in paraformaldehyde, followed by dehydration through a graded ethanol series and xylene. The tissues were then embedded in paraffin. For Masson's trichrome staining, paraffin sections were dewaxed, rehydrated, and stained according to the standard protocol. Briefly, the sections were treated with mordant solution and then stained sequentially with lapis lazuli blue dye solution, Mayer's haematoxylin, acid fuchsin-ponceau S solution, phosphomolybdic acid, and finally aniline blue dye solution. After staining, the sections were dehydrated through a graded ethanol series, air-dried, and mounted with neutral balsam. All samples were examined and imaged using an optical microscope (Leica, Germany). Subsequent image analysis was performed using ImageJ software.

### 2.5 Mitochondrial ultrastructure analysis

The collected tissues were trimmed into approximately 1 mm^3^ blocks and immediately fixed in 4% glutaraldehyde. After three rinses with phosphate-buffered saline (PBS), the samples were post-fixed in 1% osmium tetroxide for 2 h, dehydrated through a graded ethanol series, and embedded in epoxy resin. Ultrathin sections were stained with 2% uranyl acetate and examined by transmission electron microscopy (TEM; Hitachi, Japan).

Mitochondrial quantification was performed in a blinded manner using ImageJ software. For each muscle, non-overlapping fields were randomly selected from longitudinally oriented myofibers, and individual mitochondria were identified according to ultrastructural criteria. Because the gastrocnemius and soleus differ in fiber-type composition, these muscles were analyzed separately; however, individual fibers were not further classified by fiber type at the TEM level. Mitochondrial abundance was quantified as the number of mitochondria per unit area rather than per individual fiber, because TEM analysis was performed on randomly selected fields rather than on fully reconstructed single fibers. Mitochondrial size distribution was determined from measurements of individual mitochondria in randomly selected fields from each group. Size-distribution plots were generated using Nano measurer software (Fudan University, China).

### 2.6 Mitochondrial complex activity

Mitochondrial proteins were extracted using a mitochondrial protein extraction kit (catalog no. G008-1-2, Nanjing Jiancheng Bioengineering Institute, Nanjing, China), and Reagent 1 and Reagent 2 referred to below are components of this kit. The activities of mitochondrial respiratory chain complexes I, II, III, IV, and V were measured using commercial assay kits (catalog no. A089-1-1, A089-2-1, A089-3-1, A089-4-1, and A089-5-1, respectively; Nanjing Jiancheng Bioengineering Institute, Nanjing, China). All procedures were performed according to the manufacturer's instructions. Briefly, skeletal muscle tissues were homogenized in Reagent 1 and centrifuged to obtain the mitochondrial fraction. The isolated mitochondrial fraction was subsequently treated with Reagent 2 to obtain mitochondrial protein lysates. Protein concentration was determined using the BCA assay. The activities of mitochondrial respiratory chain complexes were measured spectrophotometrically using a microplate reader (BioTek, USA), normalized to mitochondrial protein concentration, and calculated according to the manufacturer's instructions.

### 2.7 ROS determination

ROS levels in skeletal muscle tissues were measured using a tissue ROS detection kit containing a dihydroethidium (DHE) fluorescent probe (BB-470515, Beibokit, Shanghai, China). Intracellular ROS levels in C2C12 cells were measured using 2′,7′-dichlorodihydrofluorescein diacetate (DCFH-DA) (BB-4705, Beibokit, Shanghai, China). For tissue analysis, the gastrocnemius and soleus muscles were frozen immediately after dissection and stored at −80 °C until use. Before analysis, the frozen tissues were briefly rinsed with ice-cold PBS to remove residual blood and connective tissue, then homogenized in ice-cold lysis buffer A (1:9, *w/v*) using a precooled tissue homogenizer on ice. The lysis buffer was a reducing agent-free system containing Tris-HCl (pH 7.5), NaCl, and Triton X-100, supplemented with protease and phosphatase inhibitors. After centrifugation, the supernatant was collected, and protein concentration was determined. All procedures were performed at 4 °C. A total of 190 μL of the supernatant and 10 μL of probe O08 were added to a black-bottom 96-well microplate and incubated at 37 °C for 30 min. Fluorescence was then measured at the 516 nm excitation and 606 nm emission according to the manufacturer's instructions. Tissue ROS levels were expressed as fluorescence intensity normalized to protein concentration.

For cell analysis, intracellular ROS levels were measured using the DCFH-DA. C2C12 cells were seeded at a density of 5 × 10^5^ cells/well. After washing twice with PBS, cells were incubated with DCFH-DA diluted in HBSS (2000:1) for 40 min, followed by PBS washing. Fluorescence images were acquired using a fluorescence microscope (Leica, Germany). For quantitative analysis, cells were collected and an aliquot of the cell suspension was transferred to a 96-well opaque microplate for fluorescence measurement using a microplate reader. Cellular ROS levels were calculated as fluorescence intensity normalized to protein concentration. Because DCFH-DA was used in this study, the cellular assay reflects total intracellular ROS rather than mitochondria-specific ROS.

### 2.8 SYW-containing serum preparation

Male SPF Kunming mice (30 ± 5 g) were randomly allocated into two groups: the control group and the SYW-treated group (2.56 g/kg). Control mice received rice soup by oral gavage according to the same schedule. After a 3-day acclimation period, mice were gavaged for 3 consecutive days with SYW at 2.56 g/kg/day, administered in two divided doses (1.28 g/kg per administration). After the final administration, the mice were anaesthetized with isoflurane, and blood was collected from the orbital venous plexus and centrifuged to isolate serum.

### 2.9 Cellular experiments

C2C12 myoblasts were cultured in Dulbecco's modified Eagle's medium (DMEM) supplemented with 10% foetal bovine serum (FBS) and 1% penicillin-streptomycin (100 U/mL penicillin and 100 μg/mL streptomycin) in a humidified incubator under standard conditions. As an organic peroxide, *tert*-butyl hydroperoxide (*t*-BHP) increases intracellular reactive oxygen species, promotes oxidative damage, and impairs mitochondrial function, and is therefore commonly used to establish an *in vitr*o oxidative injury model.^26 27^Based on the preliminary viability screening, treatment with 300 μmol/L t-BHP for 4 h reduced C2C12 myoblast viability to approximately 50% of the untreated control level, and this condition was selected for subsequent cellular experiments (Fig. S2). When cells reached approximately 60% confluence, they were pretreated with or without *t*-BHP for 4 h and then exposed to medium containing 0%, 10% or 20% SYW-containing serum for 24 h. After treatment, cells were collected for subsequent analysis.

SR18292 (HY-101491, MedChemExpress, New Jersey, NJ, USA) is a small-molecule compound used to perturb PGC-1α-related signaling and has been reported to reduce PGC-1α-dependent transcriptional coactivation.^26^^,^^27^ In the present study, C2C12 myoblasts were treated with SR18292 for 48 h, followed by treatment with SYW-containing serum for another 24 h before sample collection.

### 2.10 Cell viability assay

The effect of SYW-containing serum on the viability of *t*-BHP-treated C2C12 myoblasts was assessed using a Cell Counting Kit-8 assay. C2C12 myoblasts were seeded into a 96-well microplate at a density of 1 × 10^4^ cells per well in DMEM supplemented with 10% FBS. The cells were then exposed to 300 μmol/L t-BHP for 4 h. After washing with PBS, the cells were treated with 10% or 20% SYW-containing serum for 24 h. The culture medium was then removed and replaced with fresh DMEM containing 10% CCK-8 reagent (HY-K0301, MedChemExpress, New Jersey, NJ, USA). After incubation under standard conditions for an additional 2 h, the optical density was measured at 450 nm to assess cell viability.

### 2.11 RT-qPCR assay

RNA was isolated from skeletal muscle tissues, and its concentration and purity were assessed before reverse transcription. cDNA was synthesized using TransScript® II Reverse Transcriptase (R223-01, Vazyme Biotech, Nanjing, China) according to the manufacturer's instructions. Quantitative real-time PCR was performed using Universal SYBR qPCR Master Mix (Q712-02, Vazyme Biotech, Nanjing, China), and relative gene expression was calculated using the 2^−ΔΔCt^ method. GAPDH was used as the reference gene for normalization based on its routine use and stable amplification performance in our experimental setting. The sequences of all primer pairs used in this study are provided in Table 1.

**Table 1:** Primer sequences used for RT-qPCR.

Gene	Primer	
	Forward	Reverse
*MuRF-1*	5′-CAGCACAACCTCTGCCGGAA-3′	5′-ATGCCGGTCCATGATCACT-3′
*Atrogin-1*	5′-CCACTCTACACTGGCAACAGCAG-3′	5′-AGGCAGGTCGGTGATCGTGAG-3′
*Myostatin*	5′-TGTAATGCGTGTGCGTGGAGAC-3′	5′-GCGCTCTGGGCAGAAGTTGTC-3′
*PGC-1α*	5′-CCAAAGGATGCGCTCTCGTTCA- 3′	5′-CGGTGTCTGTAGTGGCTTGACT- 3′
*Nrf2*	5′-GTCACTCCGTGAGGCCAACCT-3′	5′-AAGTACAAACTTCCTGCCATC-3′
*HO-1*	5′-TCTTCACCTTCCCCAACATT-3′	5′-TCCTGCAACTCCTCAAAGAG-3′
*NQO1*	5′-CATGGCGGTCAGAAAAGCAC-3′	5′-ATGGCATACAGGTCCGACAG-3′
*GAPDH*	5′-GTCTCCTCTGACTTCAACAGCG-3′	5′-ACCACCCTGTTGCTGTAGCCAA-3′

### 2.12 Immunoblotting

Tissue and cellular proteins were extracted using ice-cold RIPA buffer supplemented with protease inhibitors, followed by centrifugation at 12,000 × g for 20 min at 4 °C. Protein concentrations were determined using the BCA assay. Equal amounts of protein were separated by SDS-PAGE and transferred onto PVDF membranes. Membranes were blocked with 5% non-fat milk in TBST for 1 h at room temperature and incubated overnight at 4 °C with the following primary antibodies: PGC-1α (1:1000, catalog no. NBP1-04676, Novus Biologicals), NDUFS3 (1:1000, catalog no. 459130, Thermo Fisher Scientific), SDHB (1:1000, catalog no. 459230, Thermo Fisher Scientific), UQCRC1 (1:1000, catalog no. 459140, Thermo Fisher Scientific), MTCO1 (1:1000, catalog no. 459600, Thermo Fisher Scientific), ATP5A (1:1000, catalog no. 459240, Thermo Fisher Scientific), OPA1 (1:1000, catalog no. 27733-1-AP, Proteintech), Mfn1 (1:1000, catalog no. 13798-1-AP, Proteintech), Mfn2 (1:1000, catalog no. 12186-1-AP, Proteintech), Drp1 (1:1000, catalog no. 12957-1-AP, Proteintech), Fis1 (1:1000, catalog no. 10956-1-AP, Proteintech), Nrf2 (1:500, catalog no. sc-365949, Santa Cruz Biotechnology), HO-1 (1:500, catalog no. sc-390991, Santa Cruz Biotechnology), NQO1 (1:500, catalog no. sc-376023, Santa Cruz Biotechnology), Atrogin-1 (1:1000, catalog no. ab168372, Abcam), MuRF-1 (1:1000, catalog no. 55456-1-AP, Proteintech), Myostatin (1:1000, catalog no. 19142-1-AP, Proteintech), and GAPDH (1:5000, catalog no. 60004-1-Ig, Proteintech). After washing with TBST, the membranes were incubated with the appropriate HRP-conjugated secondary antibodies for 1 h at room temperature. Protein bands were visualized using an enhanced chemiluminescence imaging system (Bio-Rad, CA, USA). Band intensities were quantified using ImageJ software and normalized to GAPDH.

### 2.13 Statistical analysis

Randomization and blinded assessment were used in the study. Sample sizes were based on preliminary data and previous SAMP8 studies, and no formal power calculation was performed. Normality and variance homogeneity were assessed before analysis. For normally distributed data with equal variances, one-way ANOVA followed by Bonferroni's multiple-comparisons test was used. For normally distributed data with unequal variances, Welch's ANOVA followed by Games-Howell multiple-comparisons test was used. For non-normally distributed data, the Kruskal-Wallis test was used. Data are presented as mean ± SEM. A two-sided p < 0.05 was considered statistically significant.

## 3 Results

### 3.1 SYW improved sarcopenia-like muscle phenotype in SAMP8 mice

Compared with age-matched SAMR1 mice, 8-month-old SAMP8 mice showed reduced hindlimb muscle volume, decreased forelimb grip strength, impaired rotarod performance, and lower spontaneous locomotor activity, consistent with sarcopenia-like muscle dysfunction. In the study, SYW was tested at 0.64 (low-dose SYW, LS), 1.28 (medium-dose SYW, MS), and 2.56 g/kg/day (high-dose SYW, HS). Among these doses, the high dose (2.56 g/kg/day) showed the most pronounced overall improvement in muscle-related phenotypes and was therefore selected for the subsequent experiments. Representative MRI images and quantitative analysis showed that 8 weeks of SYW treatment by oral gavage increased hindlimb muscle volume in SAMP8 mice (Fig. 1A and B). In addition, SYW significantly improved forelimb grip strength, rotarod performance, and spontaneous locomotor activity (Fig. 1C–E), indicating an overall protective effect on sarcopenia-like muscle dysfunction.

**Fig. 1:**
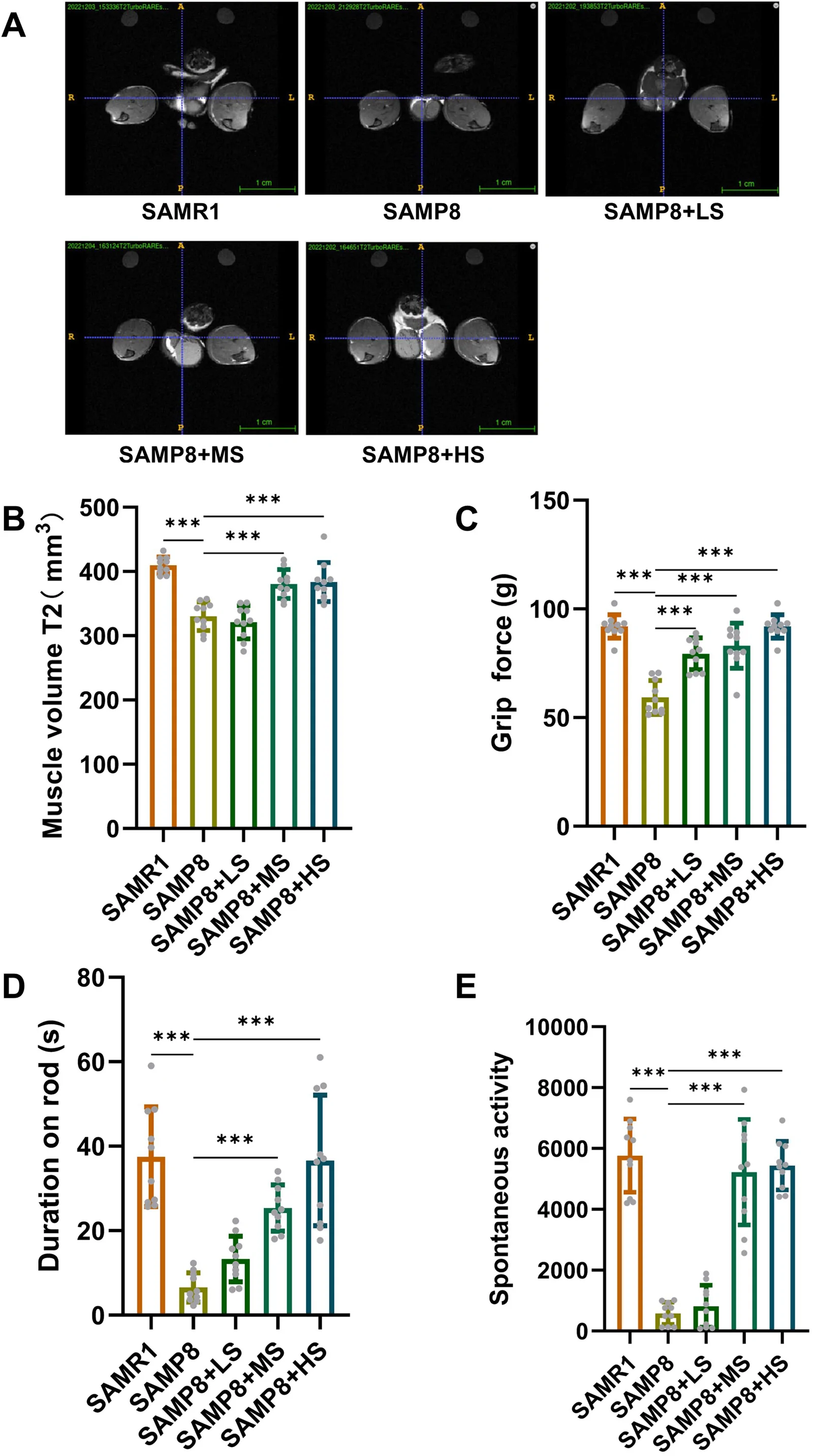
Shanyu Wan (SYW) improved muscle phenotype in senescence-accelerated mouse prone 8 (SAMP8) mice. (A) Representative magnetic resonance imaging (MRI) cross-sections of hindlimb muscles from senescence-accelerated mouse resistant 1 (SAMR1), SAMP8, SAMP8 treated with low-dose SYW (SAMP8+LS, 0.64 g/kg/day), medium-dose SYW (SAMP8+MS, 1.28 g/kg/day), and high-dose SYW (SAMP8+HS, 2.56 g/kg/day). Scale bars = 1 cm. (B) Quantification of hindlimb muscle volume measured by MRI. (C) Forelimb grip strength. (D) Rotarod performance, expressed as duration on the rod. (E) Spontaneous locomotor activity. Data are presented as means ± SEM with individual data points shown (n = 10 mice per group). *p < 0.05, **p < 0.01, ***p < 0.001.

### 3.2 SYW attenuated fibrotic and atrophy-related alterations in skeletal muscle

Masson's trichrome staining revealed increased collagen deposition and disrupted muscle architecture in the gastrocnemius and soleus muscles of SAMP8 mice compared with age-matched SAMR1 controls. After SYW treatment, collagen accumulation was visibly reduced, and muscle fiber organization was partially improved (Fig. 2A). Quantitative analysis confirmed that the collagen volume fraction was significantly increased in both muscles of SAMP8 mice and significantly decreased after SYW treatment (Fig. 2B), indicating attenuation of fibrosis-related alterations in aging skeletal muscle.

**Fig. 2:**
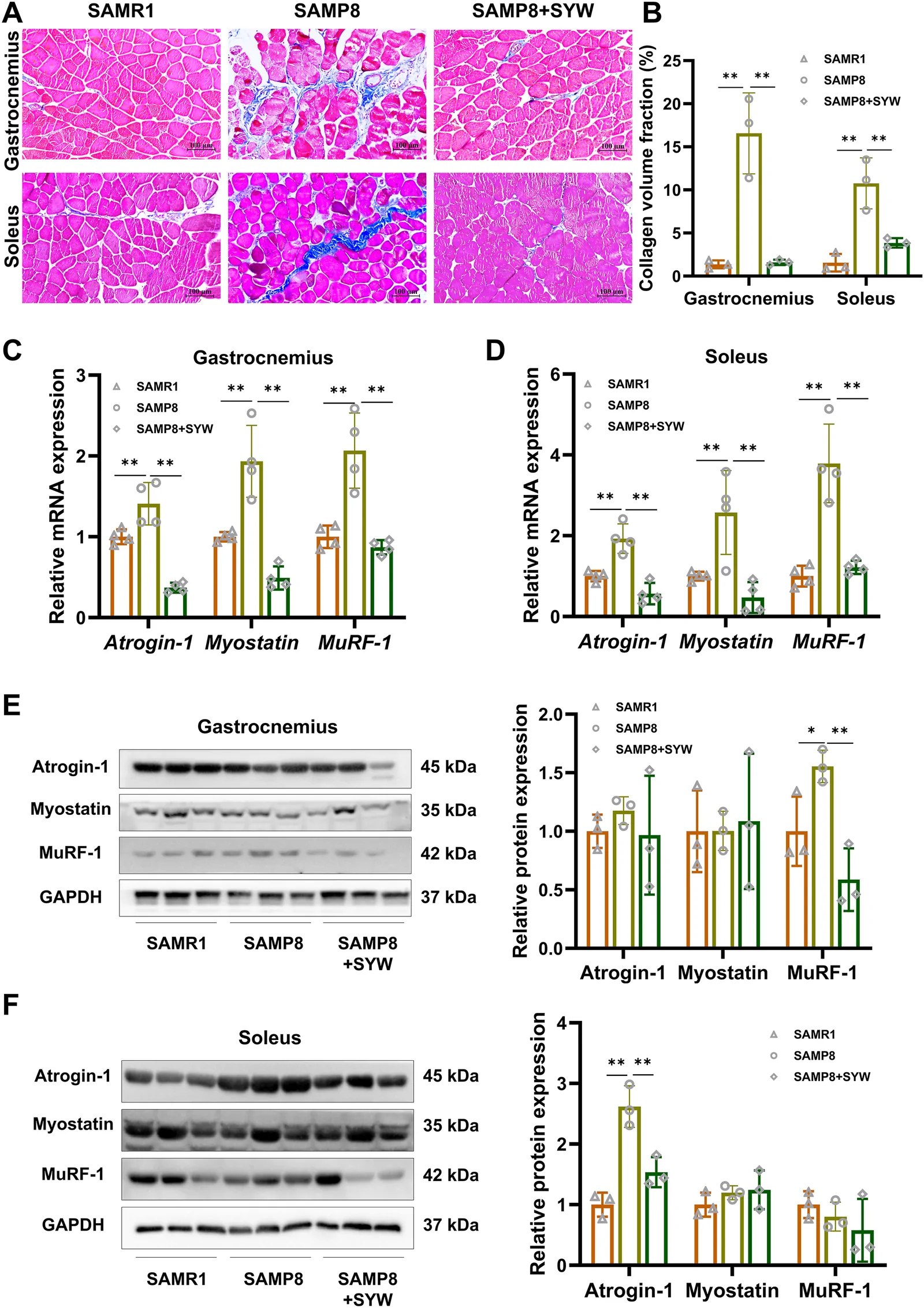
SYW treatment alleviated histopathological and atrophy-related molecular alterations in skeletal muscle. (A) Representative Masson's trichrome staining images of gastrocnemius and soleus muscles from SAMR1, SAMP8, and SAMP8+SYW mice (n = 3). Collagen fibers are stained blue and muscle fibers are stained red. Scale bar = 100 μm. (B) Quantification of collagen volume fraction in gastrocnemius and soleus muscles (n = 3). (C, D) Relative mRNA expression levels of Atrogin-1, Myostatin, and MuRF-1 in gastrocnemius and soleus muscles, respectively (n = 4). (E, F) Representative immunoblots and corresponding quantitative analysis of Atrogin-1, Myostatin, and MuRF-1 protein expression in gastrocnemius and soleus muscles, respectively (n = 3). Protein expression levels were normalized to GAPDH. Data are presented as means ± SEM with individual data points shown. *p < 0.05, **p < 0.01.

We next examined the expression of atrophy-related factors in the gastrocnemius and soleus muscles. At the mRNA level, *Atrogin-1*, *Myostatin*, and *MuRF-1* were significantly upregulated in both muscles of SAMP8 mice, whereas SYW treatment significantly reduced their expression (Fig. 2C and D). At the protein level, a significant reduction after SYW treatment was observed for MuRF-1 in the gastrocnemius and Atrogin-1 in the soleus, whereas changes in Myostatin protein were not significant (Fig. 2E and F). These findings suggest that SYW attenuates fibrosis and partially modulates atrophy-related molecular alterations in aging skeletal muscle.

### 3.3 SYW improved mitochondrial ultrastructure and mitochondrial function in skeletal muscle

Given the crucial role of mitochondrial dysfunction in sarcopenia, we next examined mitochondrial ultrastructure and function in skeletal muscle. Transmission electron microscopy (TEM) revealed evident mitochondrial ultrastructural abnormalities in SAMP8 mice, characterized by swelling, irregular morphology, cristae disruption, and reduced matrix electron density, with more pronounced changes in the gastrocnemius than in the soleus muscle. SYW administration partially restored mitochondrial morphology in both muscles (Fig. 3A and B). Quantitative analysis further showed that mitochondrial number per unit area was significantly reduced in the gastrocnemius and showed a decreasing trend in the soleus of SAMP8 mice, while mitochondrial size-distribution patterns were altered in both muscles. These changes were partially improved after SYW treatment (Fig. 3C and D).

**Fig. 3:**
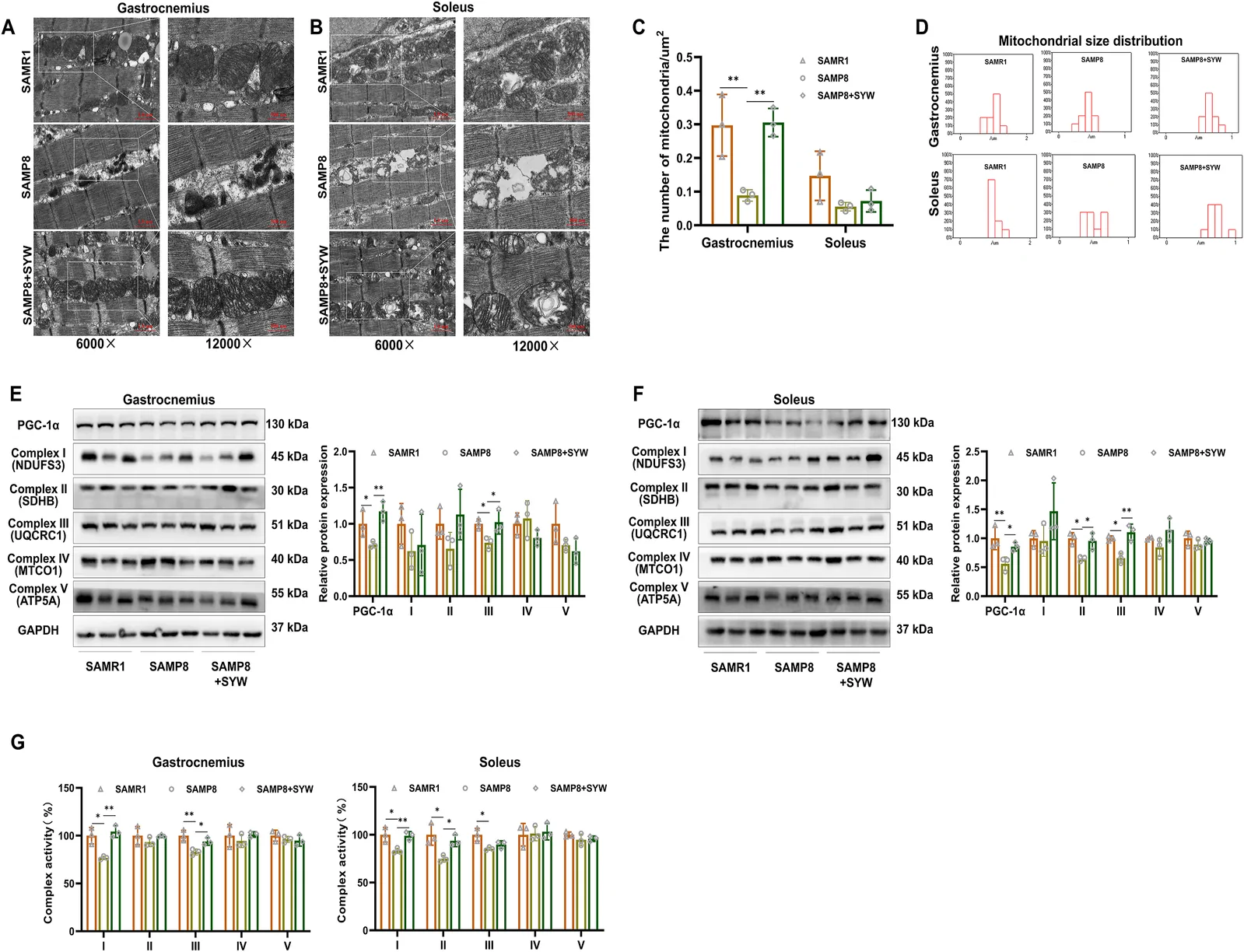
SYW treatment improved mitochondrial ultrastructure and function in skeletal muscle. (A, B) Representative transmission electron microscopy (TEM) images of mitochondria in the gastrocnemius and soleus muscles from SAMR1, SAMP8, and SYW-treated SAMP8 (SAMP8+SYW) mice at 6000× and 12000× magnification. (C) Quantification of mitochondrial number per unit area in the gastrocnemius and soleus muscles. (D) Mitochondrial size-distribution patterns in the gastrocnemius and soleus muscles. (E, F) Representative immunoblots and corresponding quantitative analysis of peroxisome proliferator-activated receptor gamma coactivator 1-alpha (PGC-1α) and mitochondrial respiratory chain complex subunits in the gastrocnemius and soleus muscles, respectively. (G) Activities of mitochondrial respiratory chain complexes I-V in the gastrocnemius and soleus muscles. Protein expression levels were normalized to GAPDH. Data are presented as means ± SEM with individual data points shown (n = 3 mice per group). *p < 0.05, **p < 0.01.

Western blot analysis further showed reduced PGC-1α expression together with decreased levels of selected respiratory chain complex subunits in SAMP8 mice. SYW treatment increased PGC-1α expression in both muscles and partially restored the expression of several respiratory chain complex subunits, particularly UQCRC1 in the gastrocnemius and SDHB and UQCRC1 in the soleus (Fig. 3E and F). Consistent with these protein changes, SYW increased the activities of complexes I and III in the gastrocnemius and complexes I, II, and III in the soleus (Fig. 3G). Together, these findings suggest that SYW partially improves mitochondrial ultrastructure and function in aging skeletal muscle, accompanied by changes in PGC-1α-related signaling.

### 3.4 SYW modulated mitochondrial dynamics and antioxidant responses in skeletal muscle

Consistent with the mitochondrial ultrastructural observations, SYW partially restored the expression of the fusion-related proteins Mfn1 and Mfn2 in skeletal muscle from SAMP8 mice, whereas OPA1, Drp1, and Fis1 showed more limited changes (Fig. 4A and B). These findings suggest that the mitochondrial protective effects of SYW may be associated, at least in part, with modulation of mitochondrial dynamics.

**Fig. 4:**
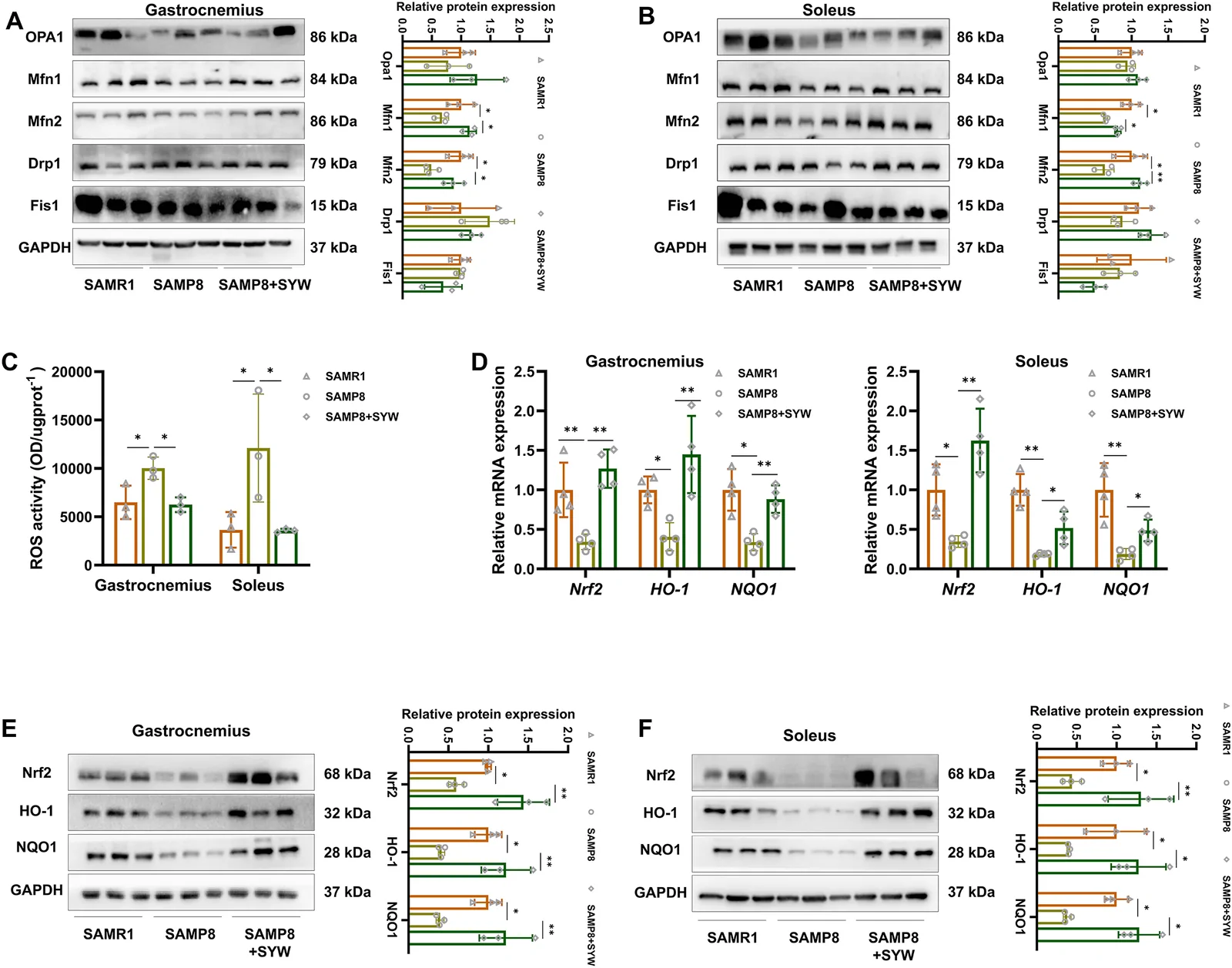
SYW treatment modulated mitochondrial dynamics and antioxidant responses in skeletal muscle. (A, B) Representative immunoblots and corresponding quantitative analysis of mitochondrial dynamics-related proteins, including optic atrophy 1 (OPA1), mitofusin 1 (Mfn1), mitofusin 2 (Mfn2), dynamin-related protein 1 (Drp1), and mitochondrial fission 1 protein (Fis1), in the gastrocnemius and soleus muscles, respectively (n = 3). (C) Reactive oxygen species (ROS) activity in the gastrocnemius and soleus muscles (n = 3). (D) Relative mRNA expression levels of nuclear factor erythroid 2-related factor 2 (Nrf2), heme oxygenase-1 (HO-1), and NAD(P)H quinone dehydrogenase 1 (NQO1) in the gastrocnemius and soleus muscles (n = 4). (E, F) Representative immunoblots and corresponding quantitative analysis of Nrf2, HO-1, and NQO1 protein expression in the gastrocnemius and soleus muscles, respectively (n = 3). Protein expression levels were normalized to GAPDH. Data are presented as means ± SEM with individual data points shown. *p < 0.05, **p < 0.01.

Compared with SAMR1 mice, SAMP8 mice exhibited significantly increased ROS levels in both the gastrocnemius and soleus muscles, which was attenuated by SYW treatment (Fig. 4C). In parallel, the mRNA and protein expression of nuclear factor erythroid 2-related factor 2 (Nrf2) and its downstream antioxidant enzymes heme oxygenase-1 (HO-1) and NAD(P)H quinone oxidoreductase 1 (NQO1) were reduced in SAMP8 mice and upregulated after SYW treatment (Fig. 4D–F). Together, these results indicate that SYW attenuates oxidative stress and enhances antioxidant defense responses in aging skeletal muscle.

### 3.5 SYW-containing serum attenuated t-BHP-induced mitochondrial and oxidative injury in C2C12 myoblasts

To further examine whether SYW exerts direct protective effects under oxidative stress conditions, C2C12 myoblasts were exposed to 300 μmol/L t-BHP for 4 h. *t*-BHP markedly reduced cell viability, whereas 20% SYW-containing serum showed a more pronounced protective effect than 10% serum and was therefore selected for subsequent *in vitro* experiments (Fig. 5A). Under these conditions, SYW-containing serum attenuated intracellular ROS accumulation, as indicated by reduced DCFH-DA fluorescence intensity and representative fluorescence images (Fig. 5B and C).

**Fig. 5:**
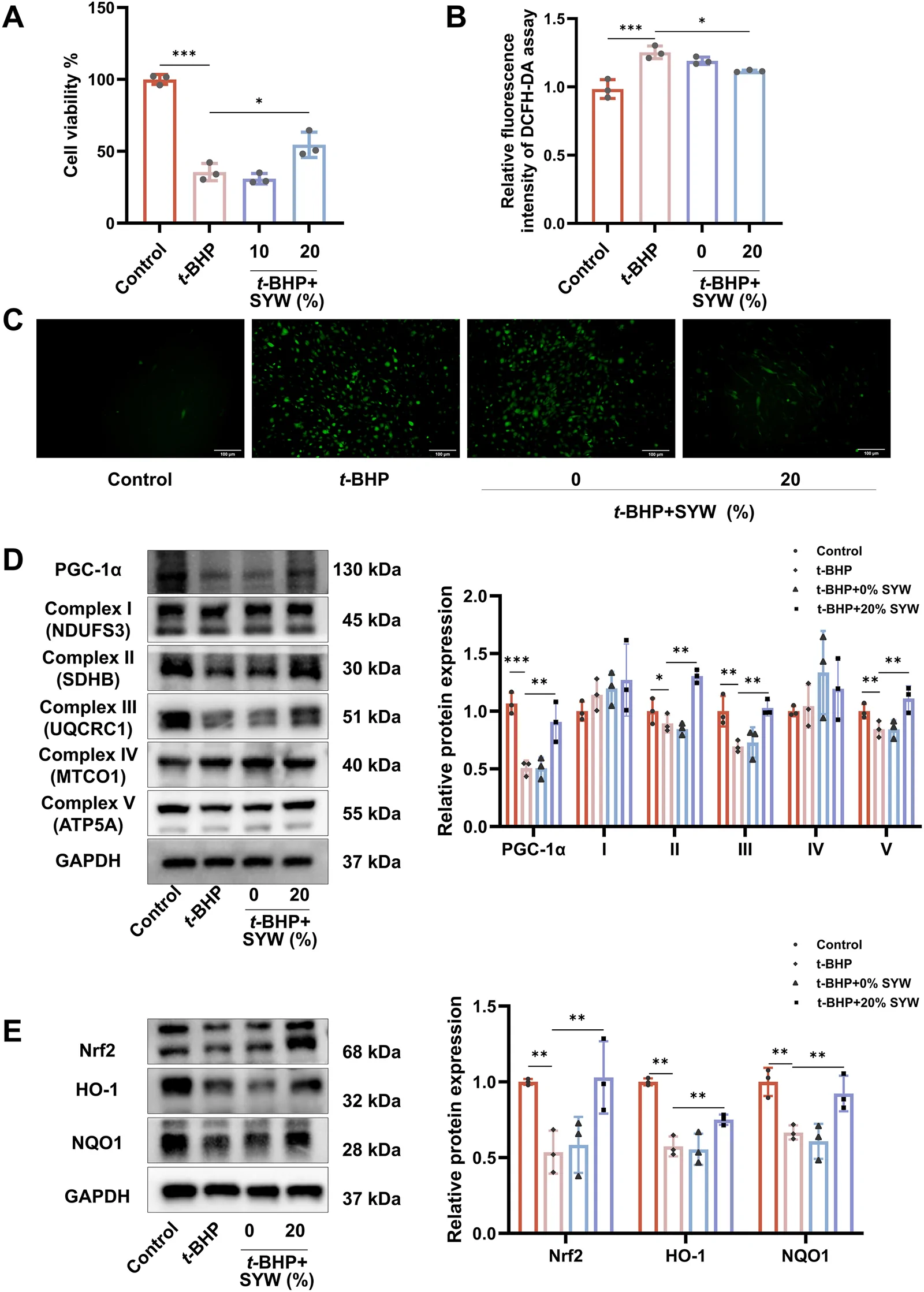
SYW-containing serum attenuated *tert*-butyl hydroperoxide (*t*-BHP)-induced mitochondrial and oxidative injury in C2C12 cells. C2C12 cells were exposed to 300 μmol/L t-BHP for 4 h, followed by treatment with 10% or 20% SYW-containing serum for 24 h. (A) Cell viability. (B) Relative 2′,7′-dichlorodihydrofluorescein diacetate (DCFH-DA) fluorescence intensity. (C) Representative DCFH-DA fluorescence images. Scale bar = 100 μm. (D) Representative immunoblots and quantitative analysis of PGC-1α and mitochondrial respiratory chain complex subunits. (E) Representative immunoblots and quantitative analysis of Nrf2, HO-1, and NQO1 protein expression. Protein expression levels were normalized to GAPDH. Data are presented as means ± SEM with individual data points shown from at least three independent experiments. *p < 0.05, **p < 0.01, ***p < 0.001.

Western blot analysis further showed that SYW-containing serum partially restored the expression of selected mitochondrial respiratory chain complex subunits, particularly complexes II, III and V, together with partial restoration of PGC-1α expression (Fig. 5D). In addition, the expression of Nrf2, HO-1, and NQO1 was increased after SYW-containing serum treatment (Fig. 5E). Together, these findings suggest that SYW-containing serum attenuates *t*-BHP-induced oxidative stress and mitochondrial dysfunction in C2C12 cells.

### 3.6 PGC-1α-related signaling is associated with the protective effects of SYW in C2C12 myoblasts

To further examine whether PGC-1α-related signaling was involved in the cellular effects of SYW, C2C12 cells were treated with SR18292, a pharmacological inhibitor of PGC-1α-related signaling. SR18292 markedly increased DCFH-DA fluorescence intensity compared with the control group, indicating enhanced intracellular ROS accumulation (Fig. 6A). Under these conditions, 20% SYW-containing serum did not reverse the increase in ROS levels.

**Fig. 6:**
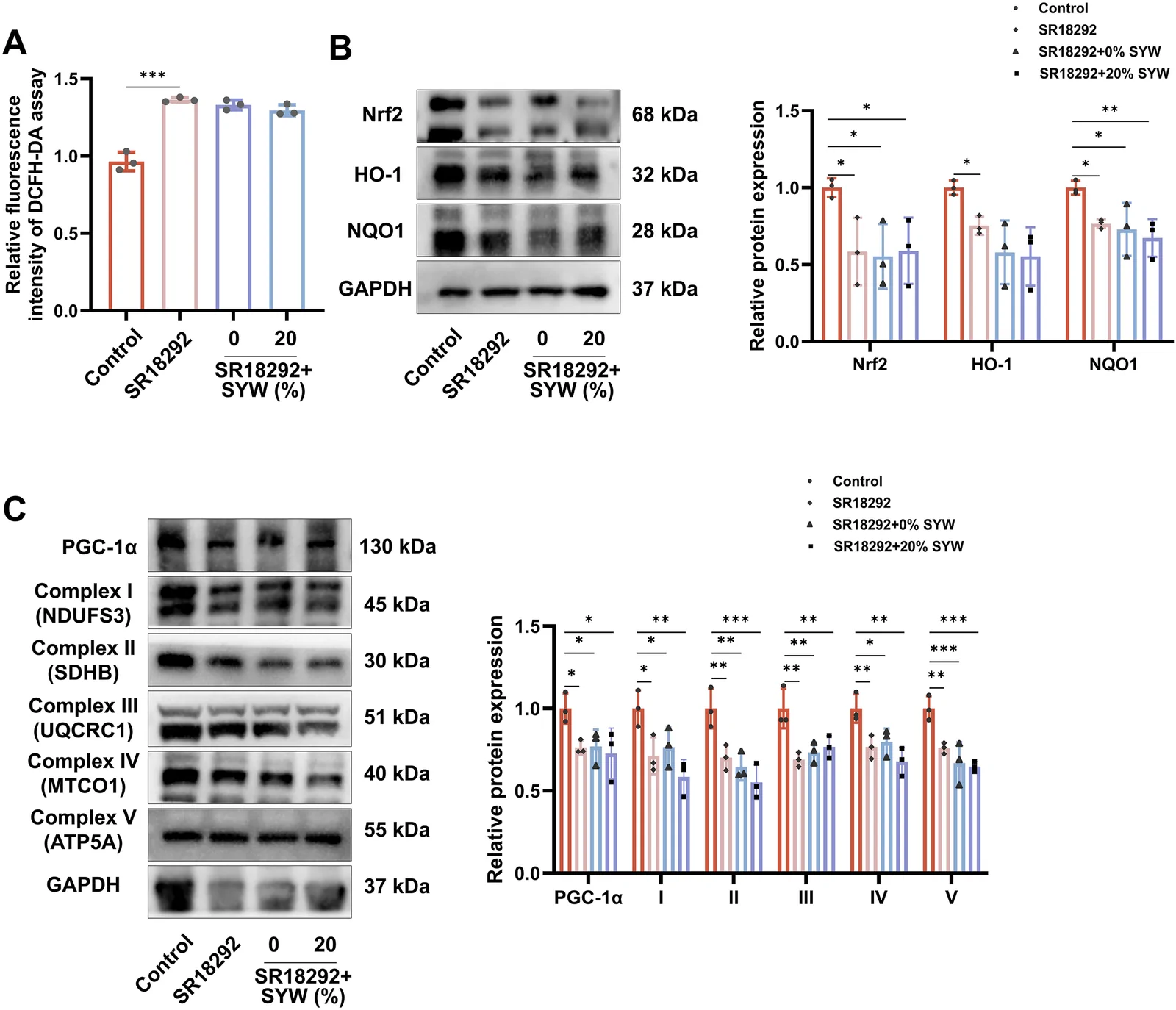
PGC-1α-related signaling is associated with the protective effects of SYW in C2C12 cells. C2C12 cells were treated with PGC-1α inhibitor SR18292 for 48 h, followed by treatment with 0% or 20% SYW-containing serum for 24 h. (A) Relative DCFH-DA fluorescence intensity. (B) Representative immunoblots and quantitative analysis of Nrf2, HO-1, and NQO1 protein expression. Protein expression levels were normalized to GAPDH. (C) Representative immunoblots and quantitative analysis of PGC-1α and mitochondrial respiratory chain complex subunits. Data are presented as means ± SEM with individual data points shown from at least three independent experiments. *p < 0.05, **p < 0.01, ***p < 0.001.

Consistently, SR18292 was associated with reduced expression of PGC-1α, mitochondrial respiratory chain complex subunits, and antioxidant-related proteins, including Nrf2, HO-1, and NQO1. SYW-containing serum did not clearly restore these changes under SR18292 treatment (Fig. 6B and C). Together, these findings suggest that disruption of PGC-1α-related signaling attenuates the cellular response to SYW, supporting the possible involvement of this pathway in SYW-mediated protection under oxidative stress. However, these pharmacological data do not establish PGC-1α as a single dominant causal mechanism.

## 4 Discussion

In this study, we found that the traditional Chinese medicine formula SYW attenuated sarcopenia-like muscle alterations in an accelerated-aging model by improving mitochondrial function and redox balance. Using SAMP8 mice and oxidative stress-induced myoblast models, we found that SYW improved muscle phenotype and reduced oxidative stress, accompanied by alterations in PGC-1α-related signaling. These findings support an association between SYW treatment and improved mitochondrial status in aging skeletal muscle.

Sarcopenia represents a multifactorial degenerative condition, and mitochondrial dysfunction has been recognized as a pivotal contributor to its progression.^28^^,^^29^ Impaired oxidative phosphorylation and excessive ROS generation disrupt protein homeostasis and exacerbate muscle atrophy.^30^^,^^31^ Our results indicate that SYW improves mitochondrial complex activity, particularly complexes I and III, supporting improved respiratory chain function in sarcopenic muscle. These findings are consistent with previous observations that restoration of mitochondrial respiration can ameliorate muscle dysfunction and delay senescence.^32^^,^^33^ Notably, the effects of SYW on myostatin were not fully consistent between the mRNA and protein levels. Although myostatin mRNA was reduced after SYW treatment, the corresponding protein change was not significant under the present conditions. This discrepancy may reflect limited or delayed regulation at the protein level, or the influence of post-transcriptional mechanisms.

An important mechanistic implication of this study is that PGC-1α-related signaling may participate in the protective effects of SYW. PGC-1α acts as a transcriptional coactivator of nuclear respiratory factors and mitochondrial transcription factor A, thereby supporting mitochondrial function and antioxidant gene expression.^34^^,^^35^ The decline of PGC-1α expression with age has been associated with reduced mitochondrial content and muscle oxidative capacity.^10^^,^^36^ In the present study, SYW was associated with increased PGC-1α expression *in vivo* and *in vitro*, whereas pharmacological interference with SR18292 was accompanied by increased ROS accumulation, and lower expression of mitochondrial respiratory chain complex subunits and antioxidant-related proteins. The SR18292 experiment supports the involvement of PGC-1α-related signaling, but the limited specificity of this inhibitor prevents exclusion of other pathways. In addition, upstream regulators of PGC-1α, including AMPK, p38 MAPK, and SIRT1, were not examined. Further pathway-specific validation is therefore required. These results support the involvement of PGC-1α-related signaling in the observed responses, but do not establish a single dominant causal mechanism.

In parallel, our data indicate that SYW is associated with activation of the Nrf2 antioxidant pathway, a downstream effector closely linked to cellular redox regulation. Nrf2 governs the expression of detoxifying enzymes such as HO-1 and NQO1, which counteract oxidative damage and contribute to redox homeostasis.^37^, ^38^, ^39^ The coordinated changes in PGC-1α-related signaling and Nrf2-related antioxidant responses suggest that SYW may exert its protective effects through multiple interacting mechanisms involving mitochondrial function and antioxidative stress regulation.

Notably, the differential effects of SYW between the gastrocnemius and soleus muscles suggest that fiber-type specificity may influence its efficacy. The gastrocnemius muscle, which contains a mixture of glycolytic white fibers and oxidative red fibers,^40^^,^^41^ showed more pronounced structural restoration and improvement in mitochondrial respiratory chain activity after SYW administration than the predominantly oxidative soleus muscle. This difference may reflect fiber-type specific regulation of mitochondrial remodeling and distinct metabolic responses between different muscle types.^42^ It is also conceivable that such heterogeneity arises from the complex pharmacology of SYW, whose multiple active constituents may differentially target metabolic and redox pathways according to fiber-type metabolic demand.

As a multi-component herbal formulation, SYW may exert its effects through the coordinated interaction of its ingredients. *Panax ginseng* has been widely reported to enhance mitochondrial function and activate antioxidant defenses.^21^^,^^43^ *Dioscorea opposita* provides polysaccharides and diosgenin derivatives that may modulate glucose utilization and muscle protein metabolism, whereas *Atractylodes Rhizoma* contributes anti-fatigue and immunoregulatory activities. The combined actions of these herbs may therefore contribute to the protective effects of SYW. However, because comprehensive phytochemical standardization was not performed and the SAMP8 model reflects accelerated rather than natural aging, the present findings should be interpreted cautiously as preclinical evidence. Further validation in naturally aged animal models and translational studies will be required.

## 5 Conclusions

In conclusion, SYW attenuated age-related muscle alterations in SAMP8 mice and improved mitochondrial function and redox balance. These protective effects were associated with changes in PGC-1α-related signaling, mitochondrial homeostasis, and antioxidant responses. Because the present study was conducted in an accelerated-aging model and the current mechanistic evidence remains at the level of pathway involvement rather than definitive causality, the findings should be regarded as preclinical evidence. Further validation in naturally aged animals and additional pathway-specific and translational studies will be required.

## Author contributions

Yue Hu: Writing-original draft, Methodology, Investigation, Formal analysis, Data curation. Qing Xiang & Cai Jiang: Methodology, Investigation, Formal analysis, Data curation. Chunxiu Huang, Huanghao Zhou, Yanyan Lan & Huijuan Wu: Software, Methodology. Zuanfang Li & Xiaodan Wang: Formal analysis, Data curation. Cong Chen: Writing-original draft, Supervision, Project administration, Conceptualization. Lidian Chen: Writing-original draft, Investigation, Conceptualization.

## Funding

This research was funded by the Joint Funds for the innovation of science and Technology, Fujian province (Grant number: 2025Y9607), Natural Science Foundation of Fujian Province (Grant number: 2023J01888).

## Declaration of competing interest

The authors declare no conflict of interest.

## Figures and Tables

**Figure:**
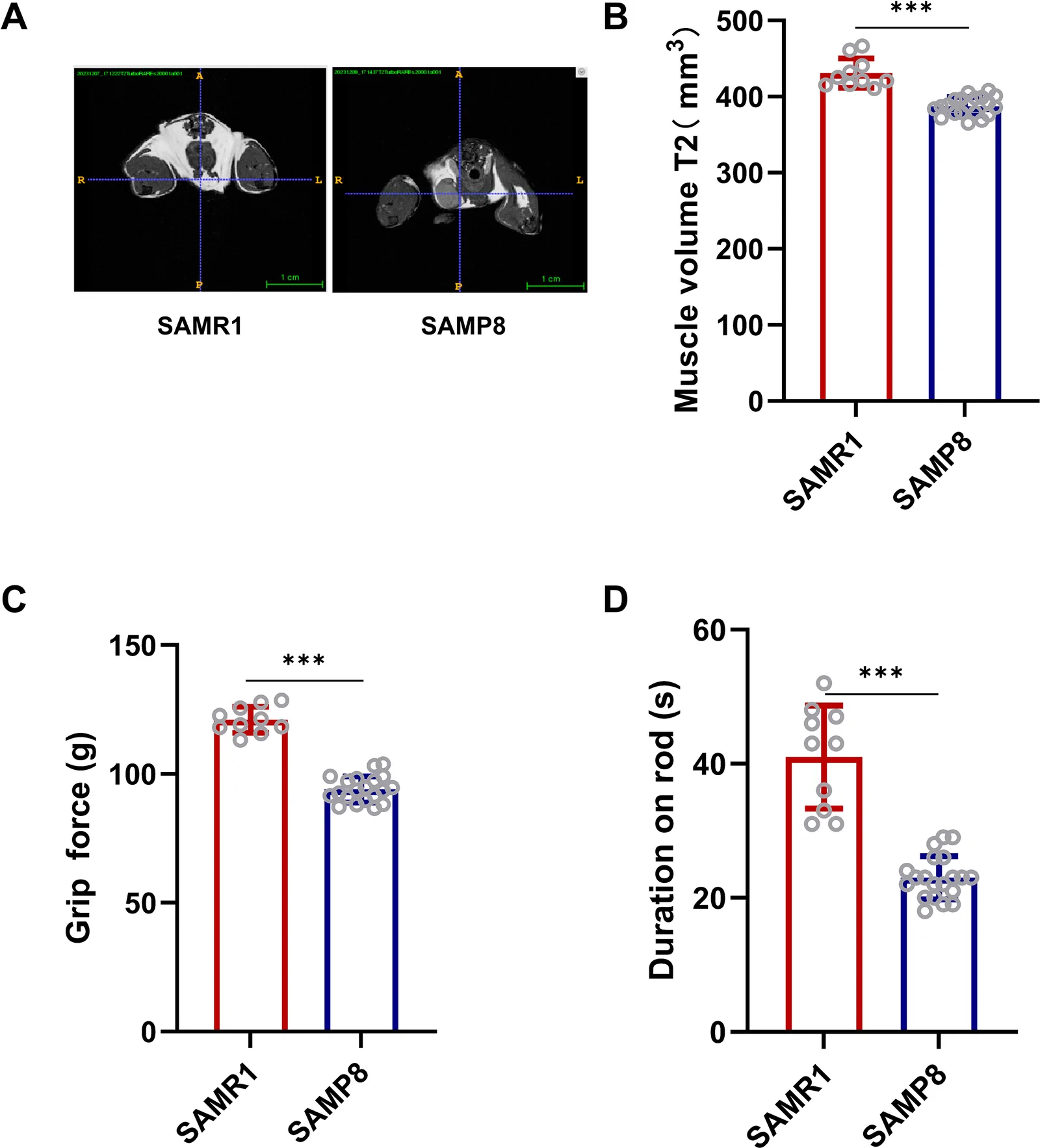
SAMP8 mice exhibit sarcopenia-like muscle alterations at 8 months of age. (A) Representative magnetic resonance imaging (MRI) cross-sections of hindlimb muscles from SAMR1 and SAMP8. Scale bars = 1 cm. (B) Quantification of hindlimb muscle volume measured by MRI. (C) Forelimb grip strength. (D) Rotarod performance, expressed as duration on the rod. Data are presented as means ± SEM with individual data points shown (n = 10 mice per group). ***p < 0.001.

**Figure:**
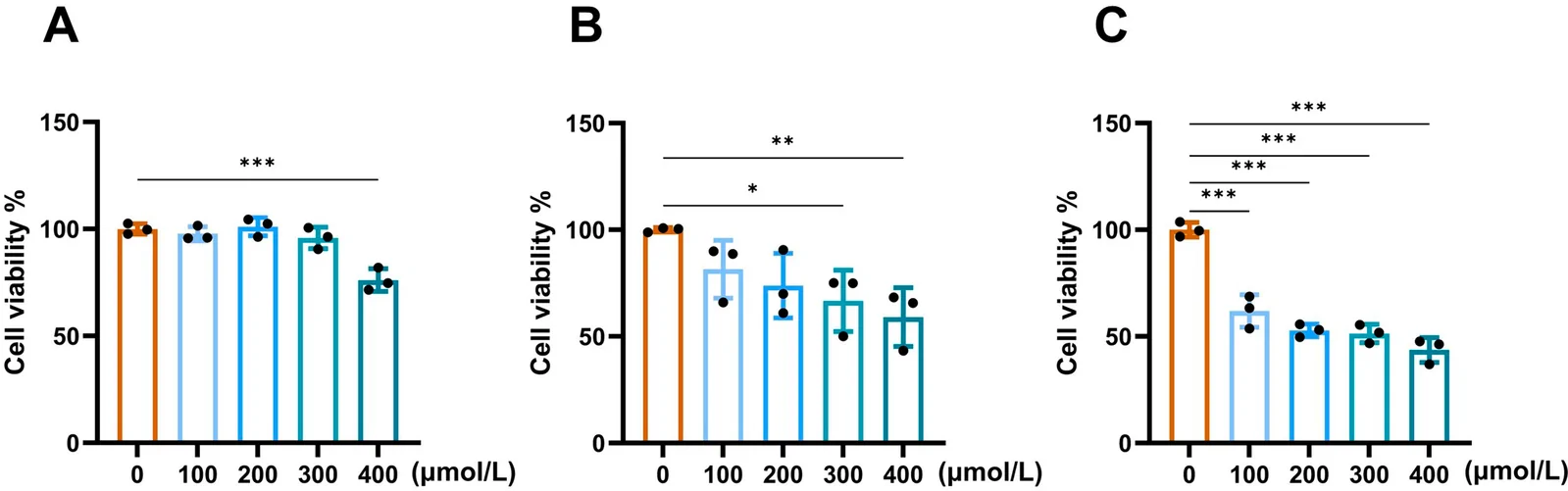
Effects of different concentrations and exposure durations of *tert*-butyl hydroperoxide (*t*-BHP) on the viability of C2C12 myoblasts. C2C12 myoblasts were treated with 0, 100, 200, 300, or 400 μmol/L t-BHP for 2 h, 3 h, or 4 h, and cell viability was assessed using the CCK-8 assay. (A) Cell viability after 2 h of *t*-BHP exposure. (B) Cell viability after 3 h of *t*-BHP exposure. (C) Cell viability after 4 h of *t*-BHP exposure. Data are presented as means ± SEM from three independent experiments. *p < 0.05, **p < 0.01, ***p < 0.001.
